# Mental Health of Frontline Nurses in India During COVID-19: A Multisite Study

**DOI:** 10.7759/cureus.55181

**Published:** 2024-02-28

**Authors:** Suja Sreedharan, Tarek Benzouak, Sanjay Rao, Farnaz N Islam, Navya Parvathareddy, Avneesh Sachdev, Swar Shah

**Affiliations:** 1 Department of Otorhinolaryngology and Head and Neck Surgery, Kasturba Medical College, Manipal, Mangalore, IND; 2 Faculty of Medicine, McGill University, Montreal, CAN; 3 Department of Psychiatry, University of Ottawa, Ottawa, CAN; 4 Department of Psychology, Carleton University, Ottawa, CAN; 5 Department of Otorhinolaryngology and Head & Neck Surgery, Kasturba Medical College, Manipal, Mangalore, IND; 6 Medicine, Royal College of Surgeons in Ireland, Dublin, IRL

**Keywords:** frontline workers, nursing, stress, somatic symptoms, personal protective equipment, depression, covid-19, anxiety

## Abstract

Background

The COVID-19 pandemic has been difficult for all healthcare providers. Nurses in Indian hospitals are at risk for mental health consequences of COVID-19-related stress. The study aimed to evaluate the mental health responses of Indian nurses working during the COVID-19 pandemic.

Method

The study was carried out during the COVID-19 pandemic from November 2020 to February 2021. Frontline nurses (n=387) working in both government and private sectors were recruited from four hospital centers across Mangalore, India. Nurses were selected based on specific inclusion criteria, including active duty within wards and intensive care units designated for COVID-19 care or suspected cases of SARS-CoV-2 infection. Recruitment and data collection were facilitated by medical residents using a mix of physical and electronic survey methods.

Results

Nurses within the private sector with low personal protective equipment (PPE) security experienced heightened anxiety. Somatic symptoms were incrementally related to mental health depending on the workplace setting; private sector staff reported greater depression symptoms compared to those in government-run hospitals. Self-efficacy buffered against depression outcomes only in nurses within the private sector working within non-COVID units.

Conclusions

This study's findings showed differential responses to the stress of COVID-19 based on the setting. Future studies should further explore the factors associated with such differences. Somatic symptoms can be indicators of mental health adversity. Early detection and supportive interventions need to be taken into account.

## Introduction

The first case of COVID-19 in India was reported on January 27, 2020 [1], and by October 4, 2021, more than 33 million COVID-19 cases had been registered [2]. Throughout the pandemic, healthcare workers have continuously played a central role in the COVID-19 response, with frontline nurses working tremendous hours to contain the outbreak [3,4]. A meta-analysis found that COVID-19 infection rates in healthcare workers are highest amongst nurses, accounting for nearly 39% of infection cases [5].

Under traditional pressures, healthcare workers represent an at-risk population with higher incidences of depression, anxiety, and stress experiences. During the pandemic, observations point to a heightened risk of these mental health adversities [6,7]. An international meta-analysis of mental health amidst the COVID-19 pandemic estimated a prevalence rate of 32% for anxiety, 40.6% for stress, and 32% for depression in nursing samples, all of which were of higher prevalence in nurses working in COVID-19 and critical care nursing environments. In addition to higher rates among nurses in charge of the care of COVID-19 patients, low self-efficacy, lack of personal protective equipment (PPE) accessibility, and the experience of physical symptoms represented major contributors to mental health outcomes [8].

Amongst a sample of 120 frontline nurses in northern India, risks of emotional exhaustion were elevated for nurses reporting low confidence in PPE and workplace safety against COVID-19 [9]. Beyond increased rates of emotional exhaustion, reports have indicated that nurses actively treating COVID-19 patients are experiencing greater levels of somatic symptoms [10]. The heightened experience of somatic symptoms in the context of nursing for COVID-19 patients has been replicated by numerous studies. For instance, an analysis of 4738 frontline nurses across 42 hospitals in China found a 42.7% prevalence of somatoform symptoms [11]. Common somatic symptoms such as sleep disturbances, fatigue, musculoskeletal complaints, and pain have high predictive values for the identification of depressive experiences [12]. Furthermore, somatic symptoms are known to be associated with other mental health experiences, such as anxiety and stress [13], and hold a dose-response association with hospital-based mental health care [14]. The heightened prevalence of somatic symptoms amongst frontline nurses may thus serve as a vulnerability for a greater risk of mental health adversities during the pandemic, although empirical testing of this has not yet been completed in the context of the COVID-19 pandemic.

In contrast to the hazardous effects of somatic symptoms, general self-efficacy has been determined to be a health-promoting factor protective of mental health adversities. Self-efficacy has been determined to predict efficient stress regulation and decreased depression [15] and anxiogenic [16] symptom experiences. Studies specific to frontline COVID-19 nurses reported a negative association between self-efficacy and anxiety experiences [17-19]. Moreover, self-efficacy is regarded as a prerequisite for effective coping with a stressor, with nurses reporting higher self-efficacy determined to be less likely to experience an acute stress disorder following exposure to a severe stressor [20]. In addition to dispositional factors, situational work-related circumstances have been demonstrated to play a pivotal role in the mental health outcomes of healthcare professionals during the COVID-19 pandemic. A recent evaluation of nurse access to suitable PPE during the COVID-19 pandemic observed that nurses who lacked adequate PPE access were 1.96, 1.64, and 1.83 times more likely to report the presence of depression, anxiety, or post-traumatic stress symptoms, respectively [21]. 

Frontline nurses across the globe have been faced with immense pressures due to the COVID-19 pandemic. Within India, it has been estimated that there is one nurse for every 2500 residents [22]. This major shortage of nursing staff has been an exacerbating factor resulting in high demands on already overworked nursing staff. A multicenter study further suggests low probabilities of mitigating the nursing shortage as 63% of nurses are estimated to emigrate due to frustrations relating to working conditions and dissatisfaction with work income [23]. Notably, nurses' experiences in India have been related to their type of employment, with nurses working in government hospitals experiencing greater job security, income, and satisfaction than their private-sector nursing counterparts [24]. However, the implications of employment types on the mental health outcomes of nursing staff during the COVID-19 pandemic remain unknown.

It is paramount to ensure the preservation and enhancement of the health of nurses. The current study aims to identify the prevalence of stress, anxiety, and depression amongst frontline nursing staff in India. Furthermore, it is expected that higher experiences of somatic symptoms, lower access to PPE, and low reports of general self-efficacy will be predictive of greater depression, anxiety, and stress experiences and higher relative risk of meeting cut-off levels for these mental health adversities. As data on the implications of COVID-19 posting remains essential to the identification of at-risk nurses amidst the COVID-19 pandemic and due to the lack of empirical reports of the implication of work type (i.e., private or public hospitals), exploratory examinations of potential moderation of these factors on the association between somatic symptoms, PPE security, and general self-efficacy and outcomes of depression, anxiety and stress symptoms will be examined.

## Materials and methods

Design and sample

This observational study was conducted within a multicenter setting using a cross-sectional design. During the November 2020 to February 2021 period of the COVID-19 outbreak, nurses were recruited from four major hospital centers in Mangalore, India, designated to provide care to COVID-19 patients or suspected cases of SARS-CoV-2 infection. Informed consent was provided through a printed document in which participants received information relating to the study, and written consent was provided by responding to a single question about understanding the risks and benefits of participation in the current study. To be included in the study, nurses needed to fulfill specific criteria related to their work settings and potential exposure to COVID-19. Eligible participants included frontline nursing staff who were actively working in areas with a high likelihood of encountering COVID-19 patients. This encompassed those serving in COVID-19 dedicated wards, intensive care units (ICUs) specifically designated for COVID-19 patients, flu or fever clinics, as well as wards and ICUs treating patients suspected of having COVID-19. Additionally, nursing staff who faced a significant risk of exposure to the virus due to their work in emergency or casualty wards and operating rooms were considered. Furthermore, the study was open to nurses working in general wards or general ICU settings, where the risk of encountering COVID-19 cases still existed, albeit at a potentially lower level compared to the previously mentioned areas. However, nurses who provided only indirect support, those with a prior history of mental health illness, or those who were unwilling to participate were excluded from the study.

Data collection

The study received ethical approval from the institutional ethics committee at Kasturba Medical College, Mangalore, and further permission to collect data from Kasturba Medical College Hospital (KMCH) Attavar, KMCH Ambedkar Circle, Lady Goschen Hospital, and Wenlock District Hospital. Data collection was initiated on November 4, 2020, and completed on February 13, 2021. Medical residents visited each hospital for recruitment and data collection. Data collection employed a self-reported survey methodology, and participants were provided with the option to respond using a physical copy or electronically using a link to a Google form version of the survey. Physical responses were transcribed electronically, resulting in the electronic storage of all anonymized responses.

Measures

Sociodemographic Information

The initial section of the survey consisted of questions concerning sociodemographic and work-related characteristics of the sample. Sociodemographics included information on participants' age, gender, medical history status, and marital status. Work-related variables included collecting data relating to the employing hospital, type of posting, and perceptions of PPE security (i.e., ranging from very bad/bad, considered as low to excellent, considered as high). As data were collected from two privatized and government-funded hospitals, hospital employment status was dichotomized as private or public. Similarly, reports of posting were dichotomized as COVID-19 setting or non-COVID-19 setting. 

Depression Symptoms

Depression symptoms were assessed using the Patient Health Questionnaire-9 (PHQ-9) [25]. The PHQ-9 is a nine-item self-rating scale for depression with questions on the frequency of depression symptoms experienced over the course of two weeks prior to participant response. Items on the PHQ-9 are measured based on a five-point Likert scale ranging from 0 to 27. A Cronbach's α coefficient of 0.89 has been reported to be associated with the PHQ-9, demonstrating a good level of internal consistency [26]. Scores obtained by the PHQ-9 scores were further dichotomized. Psychometric studies recommend a cut-off of 10, which has demonstrated a sensitivity ranging from 74% to 88% and a specificity of 84% to 91% when examining major depression disorders [25,27]. 

Anxiety Symptoms

The seven-item version of the Generalized Anxiety Disorder Scale (GAD-7) [28] was used to examine anxiety symptoms. The GAD-7 examines the frequency of anxiety symptoms within two weeks prior to participant response. Responses to the GAD-7 use a four-point Likert scale, and the total sum scores range from 0 to 21. The internal consistency of the GAD-7 has been demonstrated to range from good to excellent, with psychometric data suggesting a Cronbach's α coefficient value of 0.80 to 0.91 [29]. The scores were also dichotomized to identify cut-off levels of anxiety symptoms. A cut-off of 10 to dichotomize anxiety outcomes measured by the GAD-7 has been determined to have a sensitivity of 82% and a specificity of 89% [28]. 

Perceived Stress

Stress was measured using the Perceived Stress Scale (PSS-4) [30]. The PSS-4 includes four items assessing perceptions of stress during the four weeks before participant response and uses a five-point Likert scale. The internal consistency of the PSS-4 has been reported to have a wide range from questionable to good (0.60≤α≥0.82) [31]. A cut-off score of equal or greater than six has been suggested to dichotomize low and high reports of perceived stress [32]. Although some studies have found α coefficients below the 0.70 cut-off level, Cronbach αvalues have been demonstrated to be linked to instrumental length, and the PSS-4 has been considered reliable for empirical evaluations of perceived stress [33].

Somatic Symptoms

The 15-item somatic symptom severity scale in Patient Health Questionnaire-15 (PHQ-15) [34] was used to examine the presence of physical symptoms within the sample. The PHQ-15 is a frequently utilized tool for measuring symptom burden, such as symptoms associated with fatigue, gastrointestinal, muscular, cardiopulmonary, and pain [35]. The PHQ-15 assesses somatic symptom severity within four weeks prior to participant response using a three-point Likert scale. The internal consistency of the PHQ-15 has been reported to be good, with a reported Cronbach's α coefficient value of 0.82 within the general population [36].

Self-Efficacy

Self-efficacy levels were determined using the General Self-Efficacy Scale (GSE-10) [37]. Comprised of 10 items, the GSE-10 examines the level of agreement with items using a four-point Likert scale. The internal consistency of the GSE-10 has been reported as excellent (Cronbach's α of 0.93). 

Statistical analyses

All data were analyzed using R version 4.0.3 (R Foundation for Statistical Computing, Austria) and the Rstudio statistical environment (version 1.3.1073; Posit Software, Boston, US) [38,39]. The p-value threshold was set as 0.05 for all tests using two-tail computations. Sociodemographics were used to describe the general characteristics of the sample and reported as percentage weights or mean values with their respective standard deviation. Because missing data are attributable to participants skipping individual items, scalar scores were prorated for participants with ≤30% missing values for scale items. Descriptive data was further used to identify the prevalence of cut-off levels of perceived stress and symptoms of depression and anxiety within the nursing sample. One-way analysis of variance was utilized to determine the association between sociodemographics and workplace characteristics with continuous outcomes of perceived stress, anxiety, and depression symptoms. A Welch ANOVA with the Games-Howell post hoc test was conducted if the homogeneity of variance assumption was not met [40]. All post-hoc comparisons were derived from Tukey HSD post-hoc comparison data.

Examinations of somatic symptoms and general self-efficacy were conducted using multivariate regressions adjusting for sociodemographics and workplace-related factors to determine the unique variance associated with continuous outcomes of perceived stress, anxiety, and depression symptoms. Logistic regression modeling was further used to identify the odds ratio associated with somatic symptoms and general self-efficacy and meeting cut-off levels of perceived stress, depression symptoms, and anxiety symptoms. Questions overlapping between the PHQ15 and PHQ9 were omitted from the PHQ-15 (i.e., PHQ-15 items 14 and 15) during the modeling of depression outcomes. Notably, violation of the general linear model assumption of linearity resulted in only logistical regression modeling for a given predictor. In contrast, homoscedasticity was corrected using the HC3 correction type [41,42]. An exploratory analysis examining the moderative effect of COVID-19 posting and hospital governance (i.e., private or government) was conducted on all linear analyses. The relative risk of meeting the PHQ-9, GAD-7, and PSS-4 cut-off criterion associated with working in a government hospital and posting within a COVID-19 setting was further computed and checked for significance using Chi-square.

## Results

Participant characteristics

In total, 397 nurses were invited to participate in the study; six were excluded due to reporting a mental illness history or failing to disclose prior mental health history, and four were excluded for failing to respond to at least one study predictor and one outcome measure. A final sample of 387 nurses participated in the current study. The sample ages ranged from 19 to 55 years (30.78±7.903), and 94.06% of participants were female nurses. More than half reported being married (55.30%). The majority of the sample reported moderate levels of PPE security (67.18%), followed by high (20.67%) and low (6.98%); 5.17% of participants chose to omit to respond to questions relating to PPE security. The majority of nurses reported working in a private hospital (75.19%), and 189 worked directly with COVID-19 patients within the sample. Nurses working within the private sector were, on average, 3.75 years older (F(1,366)=16.146, p<0.001), more likely to be female (OR=3.64, X^2^=7.46, p=0.006), and report being married (OR=2.10, X^2^=9.62, p=0.002) when compared to nurses working within governmental hospitals. Sample descriptives relating to stress, self-efficacy, depression, anxiety, and somatic symptoms are presented in Table 1. 

**Table 1:** Descriptive statistics associated with perceived stress, self-efficacy, depression, anxiety, and somatic symptoms ^a^ PSS-4 - Perceived Stress Scale (perceived stress); ^b^ PHQ-9 - Patient Health Questionnaire-9 (depression symptoms); ^c^ GAD-7 - Generalized Anxiety Disorder Scale (anxiety symptoms); ^d^ PHQ-15 - Patient Health Questionnaire-15 (somatic symptoms); ^e^ GSE-10 - General Self-Efficacy Scale (self-efficacy)

Variable	Mean	SD	Median	IQR	Missing
PSS-4^a^	7.034	3.892	8	6,8	4
PHQ-9^b^	3.965	4.406	3	0,6	0
GAD-7^c^	2.795	3.799	1	0,5	0
PHQ-15^d^	5.042	4.721	4	1,8	2
GSE-10^e^	28.350	8.269	30	23,35	3

Sociodemographics and work-related characteristics were examined in relation to perceived stress, symptoms of anxiety, and depression scores (see Table 2). Notably, age was not significantly associated with depression symptoms (F(1, 366)=0.1186, p=0.742), anxiety experiences (F(1,366)=3.6894, p=0.055), and perceived stress (F(1,362)=1.3765, p=0.241). The majority of the sample met the cut-off level for severe perceived stress (72.06%), 10.08% of participants reported moderate to severe levels of depression, and 4.39% reported moderate to severe anxiety symptoms. Working within a COVID-19 setting (RR=1.2074, X^2^=8.53, df=1, p=0.0035 ) or a public hospital (RR=1.2593, X^2^=10.9, df=1, p<0.001) was determined to increase the relative risk of attaining high-stress levels. These work-related characteristics were not a determinant of moderate to severe cut-off levels of depression or anxiety symptoms.

**Table 2:** Demographical and work-related sample characteristics * p<0.05; ** p<0.01; *** p<0.001 ^a^ public hospitals: Lady Goschen and Wenlock hospitals; ^b^ private hospitals: Kasturba Medical College (KMC) Attaver and Ambedkar Circle Hospitals; ^c^ Welch ANOVA with Games-Howell post hoc test PSS-4 - Perceived Stress Scale (perceived stress); PHQ-9 - Patient Health Questionnaire-9 (depression symptoms); GAD-7 - Generalized Anxiety Disorder Scale (anxiety symptoms)

Variable		n (%)	Missing	PHQ-9	GAD-7	PSS-4
Gender	Male	17 (4.39)	6	F(1, 379)=0.1733, p=0.677	F(1,379)=0.0618, p=0.804	F(1,375)=4.0824, p=0.044^*^, M_D_=1.0681
	Female	364 (94.06)				
Marital status	Single	166 (42.89)	7	F(1,378)=0.5665, p=0.452	F(1,313)=4.3051, p=0.039^*^, M_D_=0.8364^c^	F(1,369)=3.388, p=0.053^c^
	Married	214 (55.30)				
Hospital employer	Private^a^	291 (75.19)	0	F(1,385)=0.4275, p=0.514	F(1,385)=0.8053, p=0.370	F(1,292)=28.457, p<0.001^***^, M_D_=-1.0059^c^
	Public^b^	96 (24.81)				
COVID-19 posting	Non-COVID-19	194 (50.65)	4	F(1,381)=1.0315, p=0.310	F(1,381)=5.292, p=0.022^*^, M_D_=-0.8908	F(1,365)=4.5615, p=0.033^*^, M_D_=-0.46689^c^
	COVID-19	189 (49.35)				

Protective personal equipment

Perceptions of PPE security (i.e., low, moderate, and high) were not significantly associated with depression symptoms (F(2,364)=2.0745, p=0.127) and perceived stress (F(2,361)=0.0729, p=0.930). However, PPE security did account for 4.04% of the variance in anxiety ((F(2,364)=7.6605, p<0.001, η²=0.04039). Tukey HSD post hoc identified the difference within the comparison between the low PPE security group and moderate (t=3.795, p_tukey_=0.006, MD=2.88993, SE=0.76148) and high PPE perceived security groups (t=3.668, p_tukey_= 0.002, MD=3.07423, SE=0.83821). This association between PPE and anxiety symptoms was further found to be robust to ANCOVA modeling controlling for sociodemographics and work-related factors (F(2,345)=5.9212, p=0.003, η²=0.03292).

**Figure 1:**
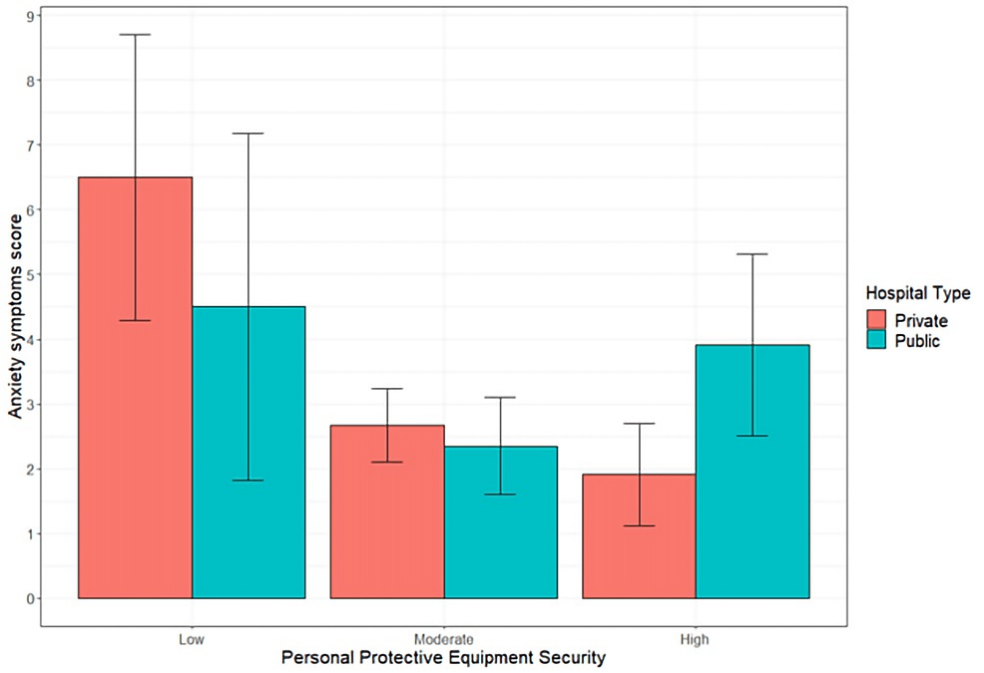
Association between PPE security and anxiety scores based on the type of hospital employment PPE - personal protective equipment

Notably, the association between PPE security and anxiety symptoms was moderated by the type of hospital employer (Figure 1). Nurses who reported low PPE security in a private hospital setting reported greater anxiety symptom levels when compared to participants reporting moderate PPE security in both private (t=2.922, p_tukey_=0.042, MD=2.888, SE=0.989) and public hospitals (t=3.074, p_tukey_=0.027, MD=3.297, SE=1.072), and nurses who reported high PPE security in private settings (t=3.518, p_tukey_=0.006, MD=3.820, SE=1.086). This moderating effect was determined to account for 2.18% of the sample's variance in anxiety symptoms (F(2,354)=4.148, p=0.0166, η²=0.022). 

Somatic symptoms

Multivariate regression modeling accounting for demographics, workplace characteristics (i.e., hospital type and COVID-19-related posting) and somatic symptoms were found to explain 28.53% of the variance associated with depression symptoms (F(10,343)=13.6947, p<0.001, R^2^=0.2853), 8.51% of the variance in perceived stress outcomes (F(10,339)=3.15368, p<0.001, R^2^=0.08511) and 29.06% of the variance in anxiety symptoms (F(10,337)=13.8044, p<0.001, R^2^=0.29059). Within all three models, only somatic symptoms were determined to be a significant predictor; uniquely accounting for 18.44% of the variance in depression symptoms (B=0.404, SE_HC3_=0.071, t=5.72, p<0.001, R^2^_part_=0.18435), 1.18% of the variance in stress perceived stress (B=0.0701, SE_HC3_=0.0230, t=3.04, p=0.003, R^2^_part_=0.01179) and 8.01% of the differences observed in anxiety symptoms (B=0.3360, SE_HC3_=0.0620, t=5.42, p<0.001, R^2^_part_=0.12933) within the sample of nurses. For each unit increase in somatic symptoms, nurses were found to be 1.21 times more likely to meet cut-off scores for depression (B=0.19753, SE(B)=0.03601, OR=1.218392, 95% CI: 1.137903-1.311220, p<0.001), 1.06 times more likely to report high perceptions of stress (B=0.05755, SE(B)=0.02773, OR=1.0592, 95% CI: 1.0049-1.1206, p=0.03799) and 1.22 times more likely to meet GAD-7 cut-off scores for generalized anxiety (B=0.19915, SE(B)=0.04670, OR=1.220361, 95% CI: 1.1172701-1.344589, p<0.001).

**Figure 2:**
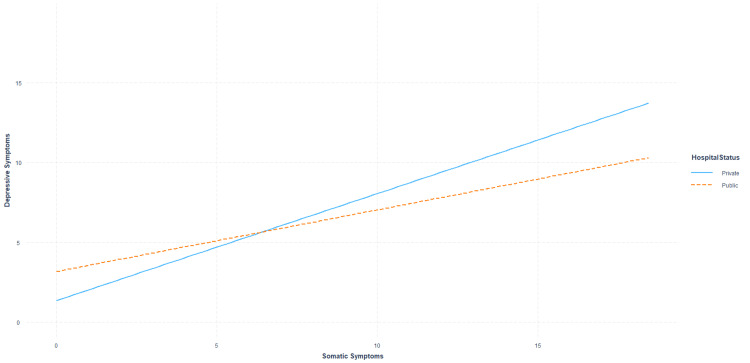
Association between somatic symptoms and depression scores based on the type of hospital employment

Notably, the association between somatic symptoms and depression outcomes was found to be dependent on the type of hospital employer. Although both nurses in private (B=0.56377, SE_HC3_=0.05622, t=10.02832, p<0.001) and public hospitals (B=0.27941, SE_HC3_=0.11153, t=2.50511, p=0.01271) were found to report greater depression symptoms as a function of somatic experiences, the association was greater amongst nurses working in private settings when considering high somatic symptom reports (see Figure 2).

General self-efficacy

**Figure 3:**
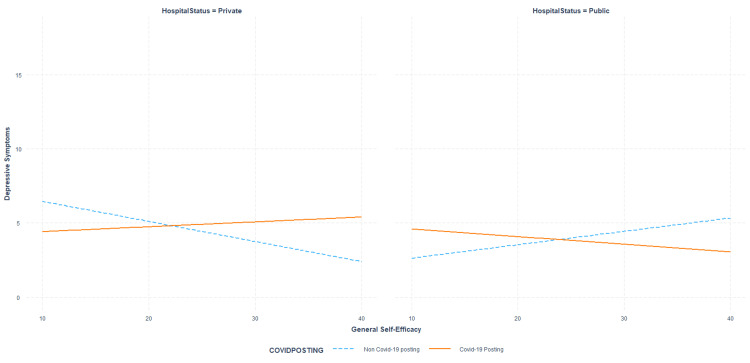
Association between general self-efficacy and depression scores based on the type of hospital employment and COVID-19 posting type

The association between general self-efficacy and depression symptoms was found to be dependent on both hospital type and COVID-19-related posting (F(10,340)=2.10655, p=0.02346, R^2^=0.05834) while controlling for sociodemographic characteristics. The three-way moderation uniquely accounted for 2.69% of the explained variance in depression reports (B=-0.07753, SE_HC3_=0.02488, t=-3.12, p=0.002, R^2^_part_=0.026899; see Figure 3). Simple slope analysis determined that the association between self-efficacy and depression only significantly differed from no change in individuals employed in private hospitals that were also working in non-COVID-19-related settings. Within this cluster, each unit increase in self-efficacy was associated with a 0.14 unit decrease in depression symptoms (B=-0.13518, SE_HC3_=0.04825, t=-2.80194, p=0.00537).

Only main effects were identified when examining anxiety and stress outcomes. Each unit increase in self-efficacy was found to be associated with a 0.03 unit decrease in perceived stress (B=-0.02573, SE_HC3_=0.01472, t=-2.39, p=0.017) and a 3.43% decreased likelihood of meeting stress cut-off levels (B=-0.03492, SE(B)=0.04670, OR=0.96568, 95% CI: 0.9375-0.99334, p=0.01767). General self-efficacy was not a significant predictor of anxiety symptoms (B=-0.03356, SE=0.03649, t=-0.91976, p=0.35835) and was not determined to be associated with any probabilistic differences in dichotomized outcomes of depression and anxiety symptoms. 

## Discussion

To our knowledge, this represents the first multicenter study examining the role of workplace characteristics, somatic symptoms, and self-efficacy as predictors of depressive, anxiogenic, and stress experiences amongst frontline nurses in India. Although high stress was higher (72.06%) than empirically reported expectations, experiences of depression (10.08%) and anxiogenic (4.39%) symptoms were lower than literature estimates of nurses' experiences during the COVID-19 pandemic [8,43]. Such geographical differences have been described in the COVID-19 literature examining community members, patients, and healthcare providers across different regions of the world [44-46]. For instance, a systematic review examining COVID-19 experiences amongst healthcare workers living in Asia reported higher rates of depression and anxiety in Chinese samples when compared to other data points on the Asian continent. In contrast, fear was determined to be of greater prevalence in non-Chinese studies when compared to data points originating from China [46].

The accessibility of PPE had been challenging during the initial phases of the COVID-19 pandemic; this had been due to the association between equipment shortages and disease rates [47]. Nonetheless, levels of PPE security have been repeatedly associated with mental health adversities and are key for the protection of nurses across the globe. As expected, perceptions of PPE security were demonstrated only to be significantly associated with anxiety experiences within the current sample. However, the association between perceived PPE security and anxiety symptoms was dependent on work-related characteristics. Low perceptions of PPE security in the private sector were linked with higher anxiety symptoms. This represents the first report of PPE-associated outcome differences among the different hospital types within the Indian healthcare system. The involvement of PPE in anxiety experiences has been suggested to involve both accessibility to PPE equipment and infection prevention training, which includes protocols to follow during a PPE breach [48]. Recent reports observed an increase in PPE accessibility and compliance in India as time progressed; however, 72% of healthcare workers reported not being aware of the procedure to undertake in the instance of a PPE breach. Evidence synthesis has demonstrated that healthcare professionals are more open to accepting occupational protections, workload reductions, and social support compared to professionally administered psychological interventions. As such, improvements to PPE access, training, and quality represent a potentially well-tolerated preventative measure to mitigate anxiety experiences among nursing staff [49]. The presence of differences between government-run and privately operated hospital centers suggests the need for further examination of differences in protocols, shortages, and training, which would promote the customization of enhancements to nurses' experiences within these differing settings.

The experience of somatic symptoms was the strongest predictor of mental health adversities within the current sample of nurses, accounting for nearly 30% of the variance in depression and anxiety symptoms and 8.5% of the variance in stress. The current study demonstrates that somatic experiences represent a robust predictor of mental health. Previous studies have shown such associations [50], but the current study has demonstrated this in the context of the COVID-19 pandemic. A scoping review inclusive of 80 papers on somatic symptoms and depression has estimated that two-thirds of patients with depression present to primary care with somatic symptoms, with each increased somatic experience representing a more substantial likelihood of depressive disorder [51]. This observation has been determined cross-culturally, with an international study estimating a prevalence of somatic experience amongst depressed patients within the range of 45 to 95% [52]. In our study, each unit increase of somatic report was associated with a 121% increase in the likelihood of meeting depressive cut-off levels. Furthermore, the association between somatic and depression symptoms was observed to depend on workplace characteristics, with nurses in the private sector reporting greater depression symptoms than nurses in government-run hospitals when somatic experiences were high. This suggests that nurses in private centers may be more vulnerable and represent an at-risk group who may be helped by frequent screening for somatic experiences and mitigation strategies to reduce depressive experiences. In contrast, anxiety and stress outcomes concerning somatic symptoms did not differ between both types of institutions.

Self-efficacy is a protective factor for mental health adversities. Higher levels of self-efficacy were observed to reduce the chances of meeting cut-off levels of stress by 3.4%. However, self-efficacy was only determined to be associated with the severity of symptomatic experiences when considering depressive experiences within the current sample. Most notably, self-efficacy was found to be dependent on both hospital type and COVID-19 posting status, in which nurses in the private sector and working in a non-COVID-19 posting were observed as the only ones experiencing buffering effects of self-efficacy in the context of depressive symptoms. This suggests that nurses working within non-COVID-19 postings in private hospitals may benefit from interventions targeting increases in self-efficacy.

Limitations

The inclusion of observations from multiple healthcare centers serves as a study strength; however, several limitations are worth considering when interpreting the results of the analysis. Although our study offers valuable cross-sectional insights into the mental health implications for nurses working in high COVID-19 exposure environments, it is inherently limited in delineating temporal dynamics. Recognizing this, there is a pressing need for the development and utilization of a longitudinal database. Such a resource would enable the tracking of nursing experiences and mental health status before, during, and after the pandemic, thereby facilitating a more precise assessment of the impact relative to baseline conditions. Future studies should consider replicating our results using longitudinal evidence.

In addition, moderation analyses were exploratory in nature and may have lacked the precision necessary to identify the presence of smaller effect sizes, although still providing preliminary evidence justifying future hypothesis-driven moderation analyses of larger samples of nurses. Notably, the sample was comprised of 94.06% females; although females represent larger proportions of the nursing workforce, future studies should aim to increase recruitment of male nurses to enhance representativeness.

All assessments of psychological or mental health experiences were conducted using vastly validated measures, representing an essential strength in the current study. However, future research should evaluate more nuanced features of certain factors. For instance, somatization in relation to patients' expression of experiences of depression or anxiogenic symptoms needs to be differentiated from somatic disorders, which hold distinct clinical markers and diagnostic implications [53]. Future research should elucidate the experience of somatic symptoms within the context of nursing during the COVID-19 pandemic, with the aim of distinguishing its presence from the expression of depressive or anxiogenic experiences or as the presence of comorbidity. In addition, there remains a need to evaluate the role of self-efficacy, employer type, COVID-19 posting, PPE security, and somatic symptoms with regard to clinical diagnostic outcomes of anxiety and depressive experiences.

Notably, healthcare systems differ across the globe, and the consideration of central aspects relating to the nursing environment should be emphasized in future research. Future studies conducted in India, for instance, should accentuate the role of center type when examining nursing experiences.

## Conclusions

Promoting and protecting the health of nursing staff represents a critical component of public health measures designed to mitigate the harms associated with the COVID-19 pandemic. The current evidence is pointing towards distinct experiential outcomes for nurses in government-run and privately operated health sectors, and future examination of specific interventions for each setting remains important to enhance the mental health of nurses during the current and any future pandemics.
